# Root Development Monitoring under Different Water Supply Levels in Processing Tomato Plants

**DOI:** 10.3390/plants12203517

**Published:** 2023-10-10

**Authors:** Oussama M’hamdi, Márton Égei, Zoltán Pék, Riadh Ilahy, Eszter Nemeskéri, Lajos Helyes, Sándor Takács

**Affiliations:** 1 Institute of Horticultural Sciences, Szent István Campus, Hungarian University of Agriculture and Life Sciences, Páter K. Str. 1, 2100 Gödöllő, Hungary; 2 Doctoral School of Plant Science, Szent István Campus, Hungarian University of Agriculture and Life Sciences, Páter K. Str. 1, 2100 Gödöllő, Hungary; 3 Laboratory of Horticulture, National Agricultural Research Institute of Tunisia (INRAT), University of Carthage, Menzah 1, Tunis 1004, Tunisia

**Keywords:** image analysis, non-destructive, root scan, root monitoring, water stress, drought

## Abstract

Managing crop yields and optimizing water use is a global challenge, as fresh water supply decreases rapidly and demand remains high. Therefore, understanding how plants react to varying water levels is crucial for efficient water usage. This study evaluates how tomato plants adapt to varying water levels (100%, 50% of crop evapotranspiration, and non-irrigated control) over two growing seasons in 2020 and 2021. Root images were captured weekly during an 8-week monitoring period in 2020 and 6 weeks in 2021 using a non-destructive CI-600 in-situ root imager at depths between 10 and 70 cm. Under water stress, plants developed deeper, more extensive root systems to maximize water uptake, consistent with prior research. Root depth and architecture varied with soil depth and the severity of water stress. Year-to-year variations were also found, likely due to changes in irrigation levels and environmental conditions such as temperature. SPAD values were higher under control conditions, especially in the 2021 growing season, suggesting reduced chlorophyll degradation, while no significant differences were observed in chlorophyll fluorescence (Fv/Fm) between treatments, suggesting stable photosynthetic efficiency under varied water stress conditions. These findings contribute to our understanding of root zone optimization and drought-resilient cultivar breeding, contributing to more sustainable agricultural practices.

## 1. Introduction

A shortage of water in the root zone is leading to reduced crop yields, and it is a major threat to global agriculture [1]. A significant factor behind this scarcity is climate change, which is forecasted to cause longer drought periods, heavier precipitation intensities, and increased evapotranspiration (ET) rates in the 21st century [2,3]. This variability in climate not only jeopardizes the sustainability of many agricultural systems but also negatively affects photosynthesis, development, nutrient uptake/accumulation, and osmotic adjustment in crops [4,5], culminating in decreased yield and quality [6].

In light of these challenges, irrigation is gaining prominence as a key adaptation strategy against drought or rainfall uncertainty. However, optimizing water-use efficiency through irrigation requires a clear understanding of the plant’s response to water stress.

Tomato (*Solanum lycopersicum* L.) is a major horticultural crop consumed and cultivated worldwide [7,8,9]. It has been chosen for this study due to its high water demand, nutritional benefits, and economic significance. The water demand of tomatoes is considerably high [10], and fluctuations in water supply can significantly impact their quality and yield [6,11]. Water deficiencies at various phenological stages also affect yield quality and quantity differently [10,12,13]. Processing tomatoes are cultivated under open field conditions and account for about 20% of the total tomato production, but their ratio is higher in the European Union [14]. It was found that different tomato genotypes may give various responses to water stress regarding root, shoot, and seedling biomass, germination, or even root length [15]. Genotype will also affect the response regarding root number and root length in tomatoes under the same level of polyethylene-glycol-induced drought stress [16]. Hence, studying the tomato plant’s response to water stress is critical for understanding its survival strategies in drought conditions. The responses of root growth to other abiotic stress factors were also studied, especially in vitro, revealing that salinity stress causes significant changes in the root caps [17], distracting the cell cycle [18].

With the advancements in technology, phenotyping methodologies have expanded significantly. These advancements provide access to better instrumentation and various methods for studying different plant parts [19,20]. Parameters such as root development and photosynthetic traits such as relative chlorophyll content (SPAD) and chlorophyll fluorescence (Fv/Fm) are now measured with non-destructive methods and studied to better understand a plant’s response to biotic or abiotic stress [21,22]. Non-destructive methods for studying root development have not been conducted on processing tomatoes yet.

To explore the soil layers and control growth orientation, plants developed a variety of tropic responses, known as tropisms, which influence the direction and size of root directional growth toward environmental cues such as gravity and water gradients. This leads to the root’s ability to seek water through hydrotropism [23], a trait especially prominent in areas with lower precipitation [24]. As a result of evaporation, the topsoil usually dries out faster, while gravity causes water to infiltrate lower soil layers, creating optimal soil conditions and directing root growth towards these deeper levels. There were cases where root growth clearly responds to a moisture gradient [24]. Comas et al. [25] reported that water is generally stored in deeper soil layers; hence, plants with deeper root systems will have access to water stored deeper in the soil.

However, the extent of hydrotropism can vary even within species [26]. Understanding these adaptations is vital for developing practices that optimize yield and water use under stress conditions [27]. For instance, research has shown that young roots tend to mature closer to the root tip when exposed to drought [28], other research has shown that the soil layer with the highest root density holds greater significance for managing water stress conditions than the absolute maximum rooting depth [29], Therefore, understanding how tomato roots respond to different water supply levels could offer insights into how the tomato plant survives under drought conditions.

Furthermore, root development is directly linked to chlorophyll content and chlorophyll fluorescence, which are essential factors for photosynthesis and plant productivity [30,31]. A well-established root system enables plants to efficiently absorb water and nutrients from the soil, facilitating the synthesis of chlorophyll molecules, while stress on the roots can impact the efficiency of photosystem II (PSII) in plants [32]. This can lead to fluctuations in the maximum quantum efficiency of chlorophyll fluorescence (Fv/Fm), depending on the genotype. Though the measurement of chlorophyll fluorescence, represented by Fv/Fm, is a useful indicator in some cases, it does not consistently correlate with drought tolerance across all crops [6]. Fv/Fm is an indirect measure of water stress, and it provides a valuable insight into the plant’s physiological status and its ability to cope with stress conditions [33].

This study aims to determine the effect of varying levels of water stress on the growth of tomato plant roots and the changes observed in specific photosynthetic traits using only non-destructive methods for plant monitoring and to examine the relationship between root development, leaf chlorophyll content, and photosynthetic activity.

## 2. Results

### 2.1. General Results Regarding Root Count and Root Length

**Figure 1:**
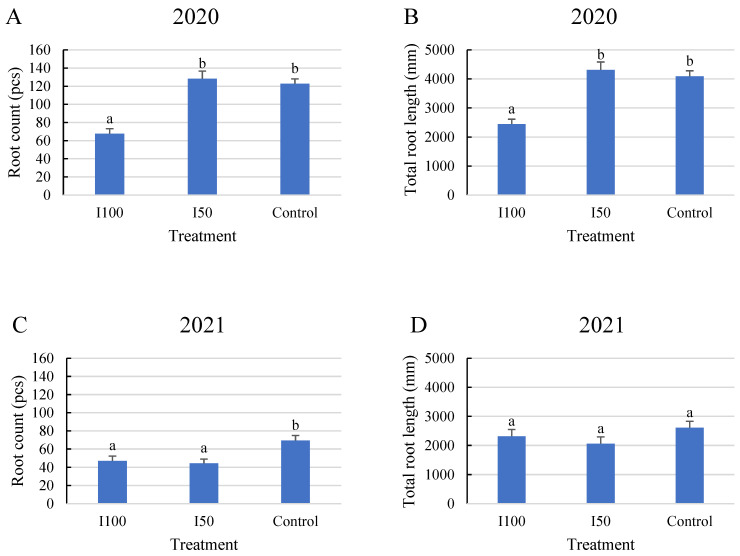
Average root count under different treatments and total root length under different treatments during the monitoring period. (**A**) Average root count in 2020, (**B**) average root length in 2020, (**C**) average root count in 2021, (**D**) average root length in 2021. Error bars represent SD. Different letters indicate statistically significant difference at *p* < 0.05 level.

In 2020, a statistical analysis showed that the full irrigation treatment (I100) resulted in a smaller number of roots with less total length than in the water-stressed treatments, meaning 45% less root per plant and 40% less total length compared to the I50 treatment and control, respectively, with no significant difference between the stressed treatments. However, plants subjected to mild and severe stress treatments developed similar average root numbers, with no significant difference. (Figure 1A,B). The results of the 2021 analysis showed that plants under the control treatment produced the highest number of roots with the highest total length, followed by the I100 and then the I50 treatments. No significant difference was found between the two irrigated treatments in 2021. Overall, fewer roots were captured in 2021 than in 2020 (Figure 1). The reason for this difference can be attributed to the different periods when a long-term irrigation deficit could develop and the different irrigation treatments that were applied. This period was determined by the meteorological conditions of the given growing season which is discussed in the Materials and Methods section.

**Figure 2:**
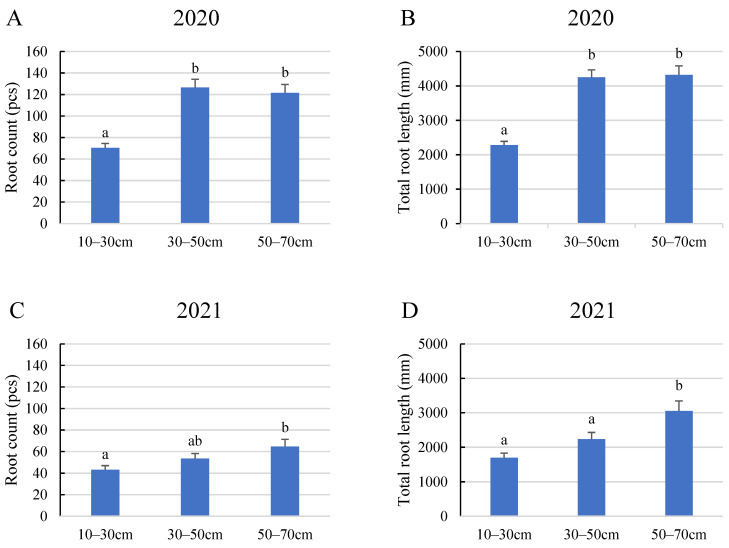
Average root count and total root length in different soil layers. (**A**) Average root count in 2020, (**B**) total root length in 2020, (**C**) average root count in 2021, (**D**) total root length in 2021. Error bars represent SD. Different letters indicate statistically significant difference at *p* < 0.05 level.

The statistical analysis of the 2020 growing season data from the perspective of layers revealed that the number and length of roots developed by tomato plants varied based on their depth in the soil. According to the results shown in Figure 2, plants generally grew more roots with a greater total root length in the middle and bottom layers (in our study, these are the 30–50 and 50–70 cm layers), featuring 127 and 122 detectable roots per plant and total lengths of 4251 and 4319 mm, respectively. In contrast, roots in the top layer did not exceed 71 roots with a 2081 mm total length. The 2021 results reinforced this pattern, indicating that root density in the soil increased with depth.

### 2.2. Evaluation of the Time Scale for the Monitored Root Zone

In 2020, the plants under the mild stress treatment (I50) exhibited significantly more roots with longer total lengths by the end of the monitoring period compared to the control and I100 treatments. Although the initial data suggested that the I50 and I100 treatments started on similar grounds, by the second week of monitoring, the rapid growth rate of the I50-treated plants led to a high root count comparable to the control. This observation could suggest that mild stress conditions stimulate the plants to develop more roots to absorb available water.

**Figure 3:**
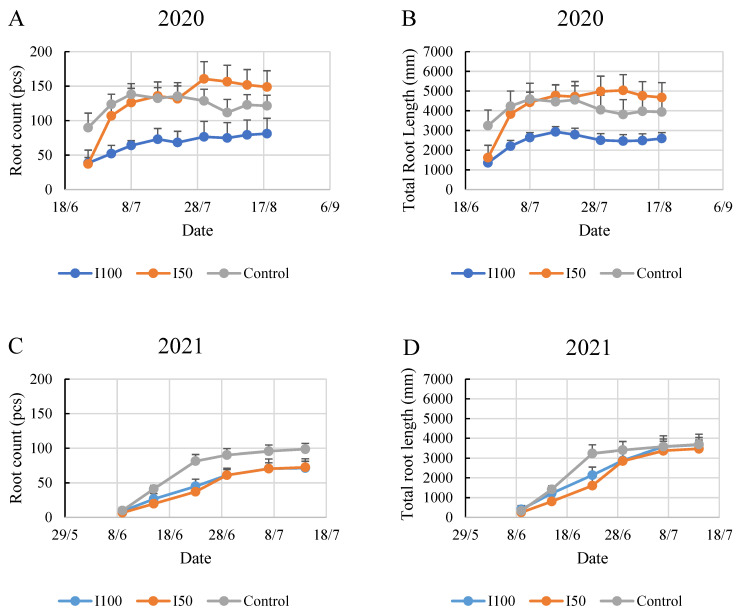
Evolution of root count and total root length in time under different water supply treatment. The numbers are summarized in the three observed root zone layers. (**A**) Evolution of root count in 2020, (**B**) evolution of root length in 2020, (**C**) evolution of root count in 2021, (**D**) evolution of root length in 2021.

In contrast to 2020, the 2021 growing season demonstrated a reduced number of roots and total root length in all the treatment groups (Figure 3). Notably, the plants in the control group showed the most extensive root growth, developing 99 roots with a total length of 3689 mm by the end of the six-week monitoring. The irrigated treatments produced similar root counts during the experiment, and the three treatments barely differed in the final two weeks of the monitoring period in root length, while the higher number of roots in the control was continuous from the second week of the monitoring period.

### 2.3. Evaluation of the Layer Scale for the Monitored Root Zone

**Figure 4:**
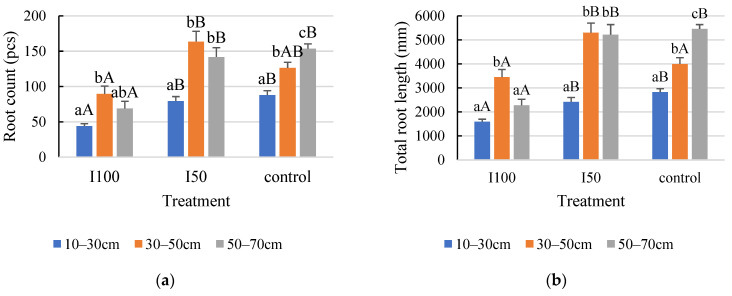
Effect of different water supply levels on root count (**a**) and total root length (**b**) across different soil layers in 2020. Error bars represent SD. Different capital letters above the columns represent statistical differences caused by different water supply levels within the same layer, while lowercase letters express differences within the same treatment between soil layers at *p* < 0.05 level.

In the 2020 growing season, the statistical analysis indicated a statistically significant difference between the layers within each treatment (Figure 4a,b). Consequently, the distribution of roots was not uniform in the 10–70 cm rooting depth. The top layer developed a smaller number of roots with the least total length under all treatments (Figure 4). Regarding the top layer, the highest number and length were captured in the control, meaning 88 roots with a length of 2825 mm in the 10–30 cm layer. The plants that received full irrigation developed the highest number and longest roots in the middle layer compared to the other two soil layers, growing 90 roots with a 3454 mm root length. The 2020 growing season data revealed no significant difference in either root number or total length between the middle and bottom layers in the mild stress treatment. Furthermore, the statistical analysis indicated that the interaction effect between treatment and layer significantly affected root length but not root count.

**Figure 5:**
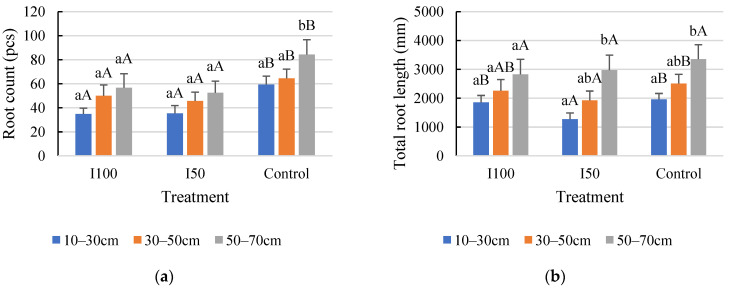
Effect of different water supply on root count (**a**) and total root length (**b**) between different soil layers in 2021. Error bars represent SD. Different capital letters above the columns represent statistical differences caused by different water supply within the same layer, while lowercase letters express differences within the same treatment between soil layers at *p* < 0.05 level.

Contrarily, the 2021 findings regarding vertical root distribution deviated from the 2020 results (Figure 5). In general, the deeper the soil layer was, the more and longer roots were developed, and the highest counts were observed in the control treatment in the 50–70 cm layer, represented by 85 roots with a total length of 3355 mm in 2021. However, the total length of roots was the lowest in the I50 treatment in the 10–30 cm layer (1272 mm), and the number of roots was equally low in the irrigated treatments in this layer (35 pcs). Comparing the number and length of roots, the data revealed that the plants in the control group developed significantly more roots in each layer compared to the irrigated treatments due to severe water stress. The results for the I50 treatment were inconsistent in the two growing seasons since the same level of root number was detected as in the I100 treatment; however, the detected roots in the 10–30 cm layer were shorter for the I50 treatment compared to the I100 treatment. This discrepancy was most remarkable in the middle layer of the I50-treated group between the two years. Additionally, the statistical analysis also showed that there is no significant interaction between treatment and layer, meaning that the impact of the water treatment on root count and total root length seems to be similar across different soil layers.

### 2.4. Root Development during the Monitoring Period

**Figure 6:**
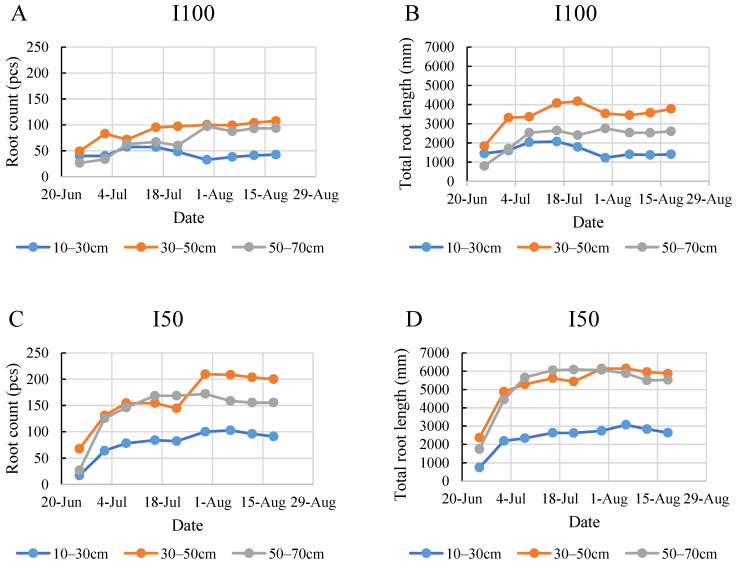

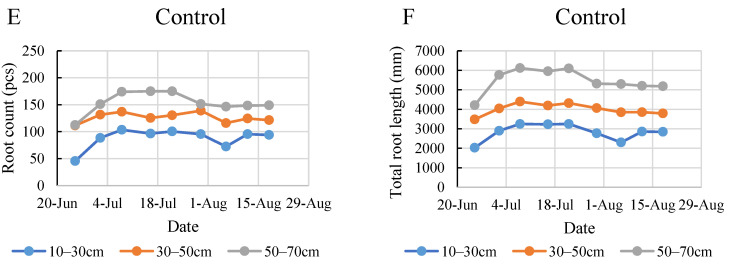
Evolution of root count (**A**,**C**,**E**) and the total root length (**B**,**D**,**F**) in different layers under the different water supply treatments in 2020.

According to the data in Figure 6, the top layer developed fewer roots under all treatments, except for the I100 treatment at the beginning of the monitoring period in the 2020 growing season. The maximum count of 104 roots in the 10–30 cm layer was found in the control group on 8 July. By the end of the monitoring period, it was found that the full irrigation treatment produced the most dense roots in the middle layer, expressed mostly in the total root length (Figure 6A,B). These graphs indicate that during the intensive root development period between 25 June and 8 July, the plants under full irrigation developed shorter root systems compared to the plants under severe and mild stress treatments. Under the mild stress treatment, the plants in the middle and bottom layers developed roots of nearly the same length throughout the entire monitoring period. The increment in root count was continuous and consistent until a later date of 30 July for the I50 treatment compared to the control and the I100 treatment, where root count stagnated after the intensive development period and even showed a decrease later in the monitoring period, especially in the control. The biggest growth in root count during the 2020 monitoring period was recorded in the 50–70 cm layer for the I50 treatment when the number of roots increased by 460% during one week (Figure 6C).

**Figure 7:**
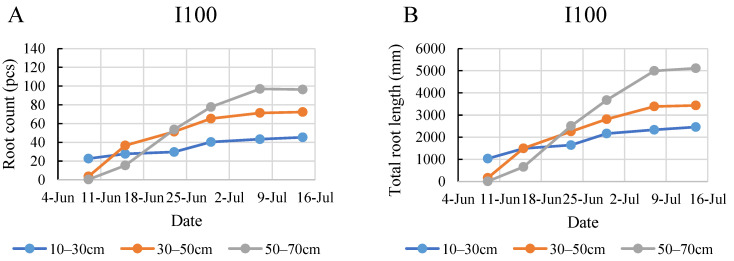

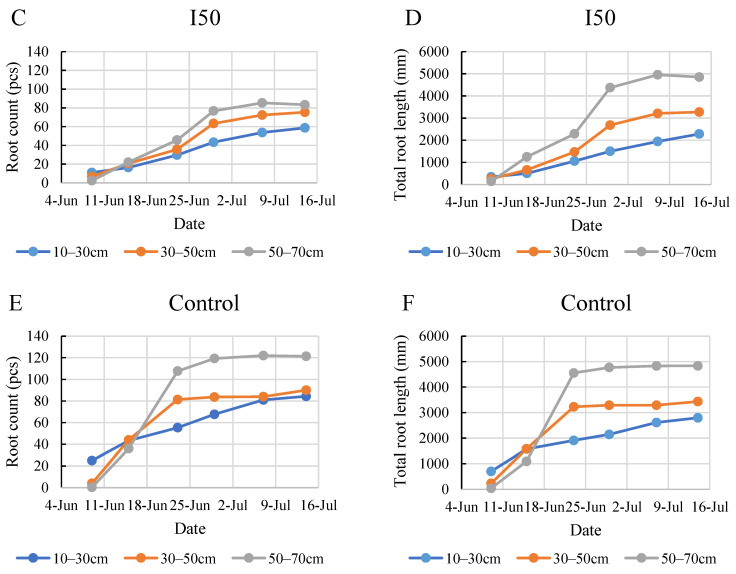
Evolution of root count (**A**,**C**,**E**) and the total root length (**B**,**D**,**F**) in different layers under the different water supply treatments in 2021.

In the initial week of monitoring in 2021, the root systems of the plants in all the treated groups were primarily concentrated in the top soil layer (Figure 7). Under the full irrigation treatment, roots showed the most robust growth in the bottom layer, reaching a maximum of 97 roots and a total length of 5113 mm. A shift was observed starting from the third week. Under all treatments, the bottom layer consistently exhibited the highest root count and total root length, followed by the middle and then the top layer. This trend became increasingly pronounced as the weeks progressed. The 50–70 cm layer became the most densely rooted layer following the period between 15 and 23 June, when the most intensive growth took place. This intensive growth period was most prominent in the control group, where the rapid development produced almost a 200% increase in root numbers and more than a 300% increase in root length. This result deviated from the 2020 findings and contradicted our expectations of denser root systems in the upper layers under frequent irrigation. Both the I50 treatment group and the control group exhibited a broader distribution of roots in the deeper soil layers sooner than in the I100 treatment group. The I50 treatment group reached a maximum of 85 roots with a total length of 4955 mm, while the control treatment group reached a maximum of 122 roots with a total length of 4835 mm. It is worth noting that, while the control treatment group demonstrated a slightly higher root count and total root length, these values seemed to stabilize in the final three weeks of the monitoring period.

### 2.5. Comparison of the Root Development in the Two Years

**Figure 8:**
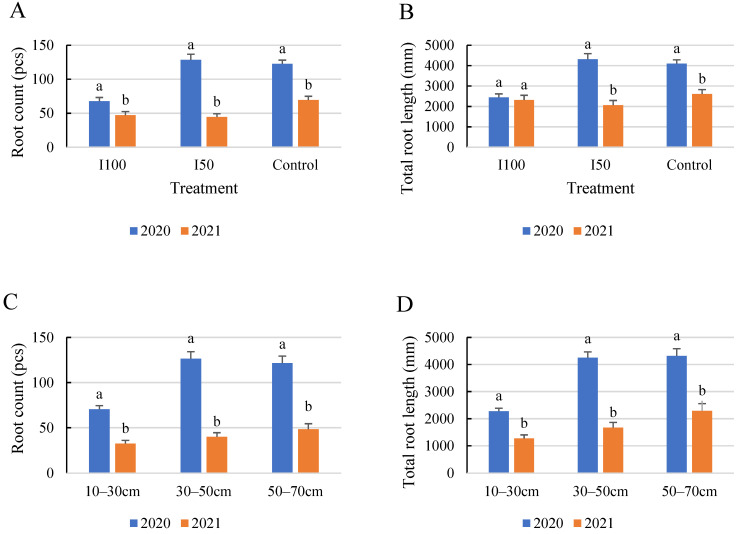
Comparison of average root count (**A**,**C**) and the average of total root length (**B**,**D**) under the different treatments (**A**,**B**) and in different soil layers (**C**,**D**) for the 2 growing seasons. Error bars represent SD. Different letters indicate statistically significant difference growing seasons at *p* < 0.05 level.

The comparative results demonstrate that the tomato plants cultivated in 2020 exhibited more substantial root growth and lengthier roots compared to those grown in 2021, regardless of the treatment applied (Figure 8). Consequently, the highest quantity and length of roots were observed in 2020 under the I50 treatment. The highest root count was recorded at 128 and 69, with corresponding total lengths of 4313 mm and 2607 mm for the years 2020 and 2021, respectively. Meanwhile, the minimum root count was observed in the 10–30 cm soil layer, with 70 and 41 roots and total lengths of 228 mm and 1610 mm, respectively, for 2020 and 2021. In 2020, the root counts were nearly equal in the 30–50 and 50–70 cm layers, whereas in 2021, both exhibited a consistent increase towards the bottom layer. The differences between treatments were less explicit in 2021.

The statistical analysis of the interaction effects between the year of measurement and the water treatment demonstrated a significant effect on root count, suggesting that the effectiveness of water treatments on root count varies depending on the year. However, for root length, a non-significant interaction between year and soil layer was observed (*p*-value = 0.115), indicating that the influence of soil layer on root length is consistent across different years.

### 2.6. Effect of Different Treatments on Relative Chlorophyll Content (SPAD) and Chlorophyll Fluorescence (Fv/Fm)

**Figure 9:**
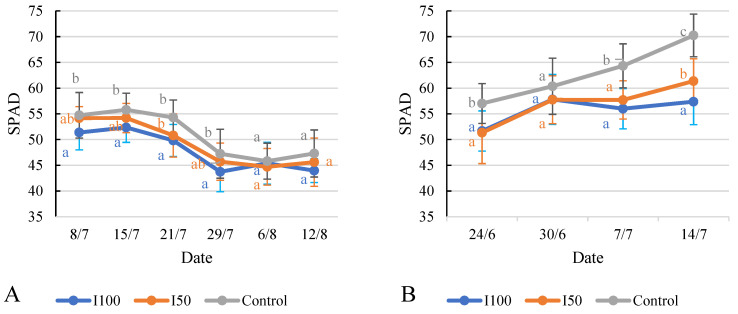
Evolution of chlorophyll content (SPAD) under different water supply treatment. (**A**) Evolution in 2020, (**B**) evolution in 2021. Error bars represent SD. Different letters indicate statistically significant difference at *p* < 0.05 level.

The available data facilitate a comparison of the SPAD values of the tomato plants under the different irrigation treatments on each measurement date. In 2020, on 8 July, the I100 treatment displayed a lower SPAD value compared to both the I50 treatment and the control treatment (Figure 9A,B). From 15 July to 29 July, the I50 treatment’s values generally surpassed those of the I100 treatment but fell short of the control treatment’s values. The SPAD values for all three treatments diminished during this period. On both 6 August and 12 August, the statistical analysis showed no significant difference between treatments. In 2021, the SPAD values of the control treatment group were significantly higher than the I100 and I50 groups during the whole measurement period, except on 30 June, where all treatments showed comparable SPAD values, indicating similar chlorophyll content. On 7 July, the control treatment exhibited higher SPAD values compared to the irrigated treatments. By 14 July, the differences between the treatments became more pronounced and significant. The I100 treatment showed a slight increase in SPAD value to 57.4, while the I50 and the control treatments exhibited a larger increase to 61.4 and 70.2, respectively, indicating a greater chlorophyll content.

Linear regression tests were performed on the means of the given treatments regarding the SPAD and the number and length of the roots. A weak relationship was found between SPAD values and root development in the 2020 growing season, where the highest regression coefficient was R^2^ = 0.22 for the root length in the 10–30 cm layer. On the other hand, in the 2021 growing season, the strongest relationship was found between SPAD values and root length in the 10–30 cm layer (R^2^ = 0.63), and the regression coefficient became lower for deeper soil layers. The linear relationship between root count in the top layer and SPAD values was even stronger, R^2^ = 0.87, and also slightly reduced with the deeper layers, but still indicated a good relationship in the deepest monitored layer, R^2^ = 0.62.

**Figure 10:**
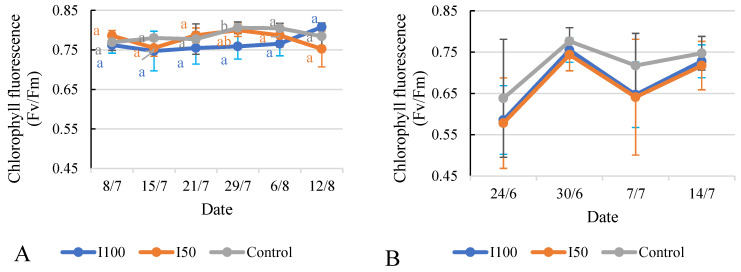
Evolution of chlorophyll fluorescence under different treatments in 2020 (**A**) and in 2021 (**B**). Error bars represent SD. Different letters indicate statistically significant difference at *p* < 0.05 level.

The data show the chlorophyll fluorescence values of tomato plants under the different irrigation treatments on each measurement date (Figure 10). In 2020, the I100 plants initially exhibited lower values compared to the I50 and control plants. However, over time, the chlorophyll fluorescence values for the I100 plants gradually increased and eventually surpassed the values of the I50 and control plants by the time of the maturity period, after the irrigation had ended. The statistical analysis indicated that, except for the measurement taken on 29 July, there were no significant differences observed between the treatments. A similar observation was recorded in 2021, when the statistical analysis revealed that there were no significant differences between the treatments on all measurement dates, except for 29 July, where the control treatment had a higher value than the I50 treatment, which reported the lowest value. Both irrigated treatments displayed very similar values.

No significant relationship was found between chlorophyll fluorescence and root development data in 2020. On the other hand, in the 2021 growing season, a similar relationship was found between the Fv/Fm data and root count in all the monitored layers, ~R^2^ = 0.35. Root length showed a similar but slightly stronger relationship, where the middle layer was found to be the highest, R^2^ = 0.41.

## 3. Discussion

The results obtained in 2020 were in agreement with the result of Scholander et al. [34], who reported that plants, when faced with water scarcity, tend to develop deeper roots, accessing sections with higher soil moisture content. Plants can avoid water-deficient conditions by maintaining relatively high tissue water potential despite a shortage of soil moisture. To maintain turgor, plants employ adaptive strategies such as increasing root depth or evolving an efficient root system to maximize water uptake [35].

Tomato plants grown under open-field conditions can endure prolonged periods of low soil water content, but the severe stress affects biomass and yield significantly [36], and a deeper root system with powerful suction force can better utilize deep soil moisture [37]. Our observations presented on Figure 5, Figure 6 and Figure 7 further support this, and they are in agreement with other studies stating that drought stress often results in a larger root system [38], which can enhance the efficacy of water uptake and assist plants in resisting water stress at the reproductive stage [39]. Previous studies have demonstrated that the frequency of irrigation not only fulfils tomatoes’ water demand but also influences the efficacy of the root system. Specifically, an increase in irrigation frequency tends to slow down root system growth; thus, larger irrigation intervals stimulate development, improving secondary root branching, main root deepening, and water and nutrient uptake [40]. According to our study, the irrigation rate affected root growth similarly to irrigation frequency, as lower irrigation rates resulted in the root system expanding to deeper layers. Root depth was also reported to increase upon exposure to water limitation in other studies, and this was confirmed by our results [41]. In addition to absorbing nutrients more efficiently, plants with deeper root systems can tolerate less frequent irrigation during subsequent growth stages [21,42,43], and as our results showed, tomato roots rapidly respond to low soil moisture conditions, promoting the deepening of the tomato root system. Previous research also demonstrated that plants adjusted their growth morphology and physiological indices to adapt to water stress over time [44]. Such structural changes could occur due to the reallocation of assimilates from the shoot to the root by plants under water stress, resulting in reduced shoot growth and enhanced root system traits such as root length and number under diminished irrigation, as water is the main driver of resource allocation [45]. Our records of rapid growth within a week reinforced this.

Root architecture may also play a significant role in water usage, as it can affect the timeline for utilizing water resources across different layers [46]. Guida et al. [47] mentioned that drought avoidance mechanisms, such as root deepening, allowed non-irrigated tomato plants to uptake water from soil layers much deeper than 40cm, which is a finding also supported by our results. This pattern aligns with previous studies [48], suggesting that roots grow downwards, especially under the 50 cm soil layer, when the upper soil profile fails to meet the crop’s water requirements. However, Schneider et al. [49] revealed that the plant’s ability to cope with water scarcity might decrease as the intensity and duration of the drought conditions increase, although other studies stated that this response can vary between different plant species and tissues [50,51]. In our case, processing tomato plants growing in dry soil during the vegetative growth stage developed deeper root systems than those growing in irrigated soil, which agrees with [43]. This was observed especially in 2021, but the roots under full irrigation were well developed in all three monitored layers, but the rate of development differed. This observation can be attributed to the increased piezometric head from the large amounts of water applied, causing moisture to move downward and promoting root system growth in the same direction, a phenomenon previously described under flood irrigation [52,53]. The results of the I50 and control treatment were more pronounced, with roots growing downward to explore available water sources, leading to more roots in the subsoil, as described by others as well [54]. A higher soil water content could enhance the growth of new roots as long as they remain aerated and below the hypoxia level [9,10,55].

Our findings from 2021 (Figure 7) showed that, for the full irrigation treatment, root development was more expressed in the upper soil layer and expanded to deeper layers over more time than for the control and I50 irrigation treatments. In another study, where the soil depth was monitored up to 2 m in wheat, it was found that the root length density in the 0–40 cm layer was the highest under well-irrigated conditions, followed by limited irrigation and no irrigation conditions [56]. The highest total root length was reached under high water conditions, supplemented with a moderate nitrogen supply, in a study conducted with minirhizotron in similar depths on cotton plants, which is in contrast with our results acquired in 2020 [57].

The difference in root growth between the two years of tomato cultivation in the same soil with the same water supply level treatment could be attributed to climatic condition differences between the two growing seasons, causing the occurrence of continuous water deficit periods attached to different phases. Temperature variations could also impact root growth, as plants tend to adapt their growth patterns in response to optimizing water uptake [58]. These findings align with other research in the field that has found that temperature and irrigation variations can significantly influence root growth patterns in various plant species [59,60].

Higher SPAD values caused by water deficiency are consistent with the findings of other studies [6], where the highest SPAD values were recorded under non-irrigated conditions during flowering and fruit settings and early fruit development. The chlorophyll fluorescence data (Figure 10) from both years indicated fluctuations in Fv/Fm values between the different irrigation treatments. Previous research has indicated that heat stress can lead to decreased maximum photochemical efficiency in detached tomato leaves [61]. Furthermore, drought-tolerant transgenic tomato plants have been found to maintain an ideal Fv/Fm value of 0.7, which indicates optimal photosynthetic performance [62]. In our study, while the Fv/Fm values varied, no significant differences were observed between treatments, suggesting that there are inherent mechanisms in tomato plants that maintain stable chlorophyll levels and photosynthetic efficiency, even under water stress conditions. These mechanisms could include adjustments in stomatal conductance, osmotic adjustment, or the activation of antioxidant defense systems that help mitigate water stress’s negative impacts on chlorophyll-related parameters [37]. In 2021, the Fv/Fm value was lower than the 2020 results, which could be due to differences in temperature between the two years. The higher Fv/Fm value in the control treatment in 2021 could indicate that plants without regular irrigation have to better adapt to environmental conditions, leading to increased photosynthetic efficiency.

While our non-destructive root monitoring method offers valuable insights, it is important to acknowledge certain inherent limitations. Due to the intricate nature of root systems, roots in close proximity might not always be distinctly analyzed. Additionally, image clarity can sometimes be compromised, potentially affecting the precision of the analysis. A notable constraint of this root scanning method was its ability to capture only the roots growing adjacent to the curved surface of the tube.

## 4. Materials and Methods

### 4.1. Plant Material and Experimental Set-Up

The experiment was conducted at the Horticultural Experimental Farm of the Hungarian University of Agriculture and Life Sciences (GPS: 47°34′51.6″ N 19°22′39.0″ E), in Gödöllő, Hungary. The field features a loamy soil, characterized by 47.5% silt, 41% sand, and 11.5% clay, with 1.6% humus content in the upper 0–30 cm layer. Processing tomato plants generally have a determined growth type and thick fruit skin. A processing tomato hybrid, H1015, was used in the experiment, which is a variety with a determinate growth type that is widely used by growers in our region. It has good adaptability to the growing conditions, and its maturity is favorable under our climate. This variety has been used in irrigation deficit experiments in other studies as well [63,64].

The row spacing was 140 cm, and the plant spacing was 20 cm, resulting in a plant density of 3.57 plants per square meter. Seedlings were transplanted on 14 May 2020 and 15 May 2021. The fertilization schedule was adjusted to the phenological phase of the tomato plants. Specific quantities of N, P_2_O_5_, and K_2_O were administered in each phase based on the plant’s needs. The total distribution for the growing season was 129 kg ha^−1^ of N, 89 kg ha^−1^ of P_2_O_5_, and 317 kg ha^−1^ of K_2_O in 2020 148 kg ha^−1^ of N, 67 kg ha^−1^ of P_2_O_5_, and 121 kg ha^−1^ of K_2_O in 2021. Plant protection interventions were applied when they were needed, according to a local expert, uniformly to all treatments.

In 2020, fertilization was carried out using a solid fertilizer, whereas in 2021, a solution was applied via drip tape with 10 cm emitter spacing in all treatments. The fertigation circuit was separate from the clean irrigation water supply, which ensured equal and uniform fertilization across all treatments.

**Figure 11:**
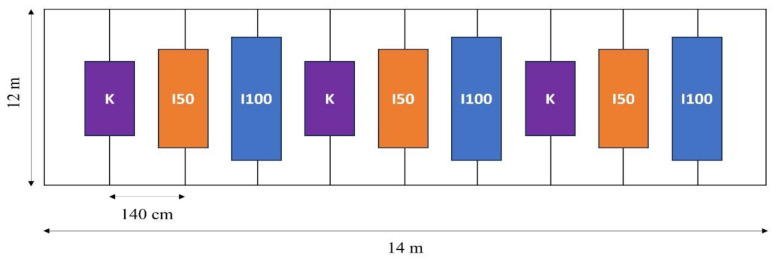
The experimental design for both experimental years.

Clean irrigation water was supplied through a drip irrigation system. Three distinct treatments were implemented, supplying 100% of crop evapotranspiration (I100), 50% of I100 (I50), and a non-irrigated control (K). Each treatment was replicated thrice to ensure the robustness of our data. As depicted in Figure 11, the experimental design ensured an even application of the different treatments. The I100 treatment received 241 mm of water, the I50 received 201 mm, and the non-irrigated K received 159 mm during the monitoring period of the first growing season. The crop evapotranspiration was 231 mm, averaging 4.2 mm per day in 2020. In 2021, the total water supplies for the I100, I50, and K treatments were 206 mm, 129 mm, and 51 mm, respectively, with 180 mm evapotranspiration, averaging 5 mm per day during the monitoring period. These data indicate that the 2021 observations occurred during a drier period.

Different types of drip tapes were employed to supply varying water quantities. A 10 cm emitter spacing tape delivering 10.6 L per hour per meter was used for the I100 treatment, whereas a 15 cm emitter spacing tape providing 5.3 L per hour per meter was used for the I50 treatment (Irritec S.p.A., Rocca di Caprileone, Italy). The positioning of the drip lines, monitoring tubes, and tomato plants remained constant across the treatments. The FAO Penman–Monteith method was used, utilizing the AquaCrop 6.1 software to calculate crop water demand, incorporating meteorological data from a nearby station [65]. The irrigation treatment in the 2020 growing season began on 8 June but was followed up only on 29 June and concluded on 3 August. In 2021, the irrigation treatment started on 3 June and ended on 2 August.

### 4.2. Image Acquisition

**Figure 12:**
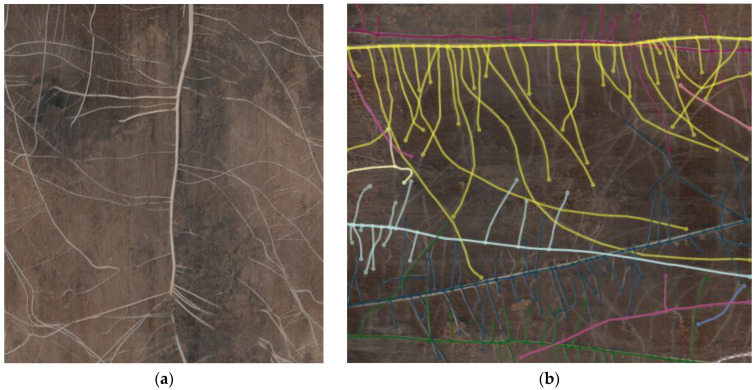
Image taken by CI-600 in situ root imager (**a**) and an image taken during the analysis process (**b**).

Images of the roots were taken using a CI-600 in situ root imager (CID Bio-Science Inc., Camas, WA, USA) (Figure 12). This device enables non-destructive imaging of living roots. During operation, the cylindrical scanner rotates within a tube featuring a transparent wall. The standard tube measures 105 cm in length, with an inner diameter of 6.35 cm and an outer diameter of 7 cm. The scanning head is 34.3 cm long, with a diameter of 6.35 cm. The resultant images measure 21.6 × 19.6 cm. Images were captured in 300 dpi resolution at three different depths: 10–30 cm, 30–50 cm, and 50–70 cm, respectively. The scanner was calibrated with a white calibration tube at the start of each measurement event. CI-600 Root Scanner 4.04 software was employed to conduct the scans. The monitoring was carried out from 25 June to 18 August, taking images once a week in 2020 and from 9 June to 14 July in 2021. Root monitoring was chiefly tied to periods when deficit irrigation treatments were continuously applied.

The scanner tubes were installed in three replications per treatment immediately following the transplantation of tomato seedlings. These tubes were set up next to randomly selected plants within the treatments. This installation method did not cause harm to the roots, as the tubes were in place prior to root development in the field.

### 4.3. Image Processing

Each loaded image was defined by the tube it was captured in, the depth in the soil, and the date of acquisition. The brightness, contrast, and gamma of the images were adjusted to achieve the highest clarity. Root distribution mapping was performed manually by tracing the cursor along the length of the root adding points, and branches were connected to the parent root. Data from RootSnap 1.4 software, concerning tubes, windows, or sessions, were exported to a spreadsheet.

### 4.4. Relative Chlorophyll Content and Photosynthetic Activity

Measurements were conducted on randomly selected plants within each treatment at around 12:00 on each measurement date. The SPAD index was measured using a SPAD 502 chlorophyll meter (Konica Minolta, Warrington, UK), And a PAM 2500 fluorometer device (Heinz Walz GmgH, Effeltrich, Germany) was used to measure chlorophyll fluorescence. Data were acquired from the device with PamWin-4 4.01 software. DLC-8 leaf clips were applied to leaves 30 min before measurement to provide dark acclimation before taking the fluorescence measurement, in which Fv/Fm values were recorded. In total, 16 measurements per treatment were taken, which included 4 measurements per treatment in each repeated block. All measurements were carried out non-destructively on healthy, fully developed leaves.

### 4.5. Statistical Evaluation

A two-way analysis of variance (ANOVA) was performed to detect the differences between root count and root length per irrigation treatment (differing levels of irrigation) and per layer (varying root depths). Then, the effect of different treatments was evaluated on the root count and total root length in different soil layers. Results were considered statistically significant at *p* < 0.05 for the Tukey test using SPSS statistics version 22.0 (IBM, Armonk, NY, USA). A linear regression was performed to reveal the relationships between SPAD values, chlorophyll fluorescence, and root count and total length data.

### 4.6. Meteorological Data

**Figure 13:**
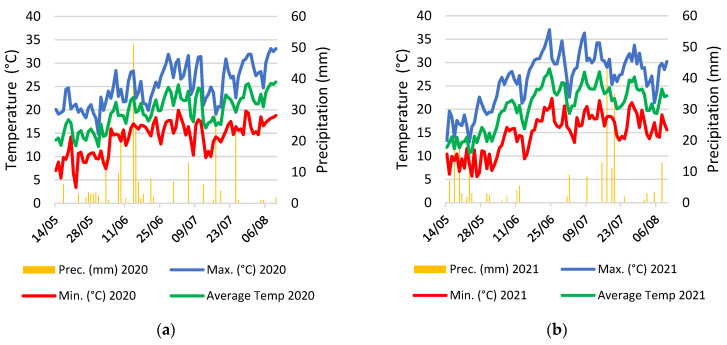
Temperature and precipitation in the 2020 (**a**) and in the 2021 (**b**) growing seasons.

The meteorological data are presented in Figure 13. The average temperature in 2021 exhibited a decline compared to 2020. However, an intriguing contrast was observed in the extremes of the thermal range. Despite a lower average, 2021 reported both higher maximum and lower minimum temperatures, underscoring a significant increase in annual thermal amplitude compared to the previous year. Regarding precipitation, the total recorded amount of 236mm for 2021 was marginally less than that of 2020 (262 mm). Noteworthy, the temporal distribution of rainfall events throughout the growing season was different, suggesting that the main rainy periods differed between the 2020 and 2021 growing seasons as they were concentrated in the middle of June in 2020 and in the middle of July in 2021 (Figure 13). At the same time, the temporal distribution was more balanced during the 2020 growing season.

## 5. Conclusions

Our study highlights the adaptability of tomato plants in response to varying water supply levels. The development of deeper roots under water stress, as observed in our findings, emphasizes plants’ inherent strategies to counter water deficits and optimize water uptake. According to our results, the root system expansion to layers with higher soil moisture levels can happen quickly (<one week). The data suggested that root length could triple in 8 days. However, tomato plants that are irrigated regularly with sufficient water quantities develop shorter roots during the intensive root development phase.

Our findings also shed light on the impact of water supply on root system efficacy, with lower irrigation rates and water quantity levels stimulating more intensive root development. The observed variances in root growth over the two consecutive years, influenced by factors such as irrigation water levels and temperature variations, underscore the multifaceted nature of plant responses to environmental conditions.

The relationship between relative chlorophyll content and root development is stronger during the intensive root development period. The consistency in chlorophyll fluorescence across treatments, despite varying water conditions, suggests robust plant mechanisms that maintain photosynthetic efficiency under stress, even if the relative chlorophyll content is affected.

Our research contributes valuable insights into the adaptive strategies of plants under drought stress. This knowledge could inform plant breeding efforts aimed at developing cultivars that are more effectively adapted to water-deficient conditions. It is also pertinent to irrigation professionals seeking to enhance the use of soil layers and improve the effectiveness of root zones.

## Data Availability

The authors confirm that the data supporting the findings of this study are available within the article.
